# Voltage-Polarity Dependent Programming Behaviors of Amorphous In–Ga–Zn–O Thin-Film Transistor Memory with an Atomic-Layer-Deposited ZnO Charge Trapping Layer

**DOI:** 10.1186/s11671-019-3204-7

**Published:** 2019-12-02

**Authors:** Dan-Dan Liu, Wen-Jun Liu, Jun-Xiang Pei, Lin-Yan Xie, Jingyong Huo, Xiaohan Wu, Shi-Jin Ding

**Affiliations:** State Key Laboratory of ASIC and System, School of Microelectronics, Fudan University, Shanghai, 200433 China

**Keywords:** ZnO, In–Ga–Zn–O, Nonvolatile memory, Thin-film transistor (TFT), Oxygen vacancy

## Abstract

Amorphous In–Ga–Zn-O (a-IGZO) thin-film transistor (TFT) memories are attracting many interests for future system-on-panel applications; however, they usually exhibit a poor erasing efficiency. In this article, we investigate voltage-polarity-dependent programming behaviors of an a-IGZO TFT memory with an atomic-layer-deposited ZnO charge trapping layer (CTL). The pristine devices demonstrate electrically programmable characteristics not only under positive gate biases but also under negative gate biases. In particular, the latter can generate a much higher programming efficiency than the former. Upon applying a gate bias pulse of +13 V/1 μs, the device shows a threshold voltage shift (ΔV_th_) of 2 V; and the ΔV_th_ is as large as −6.5 V for a gate bias pulse of −13 V/1 μs. In the case of 12 V/1 ms programming (P) and −12 V/10 μs erasing (E), a memory window as large as 7.2 V can be achieved at 10^3^ of P/E cycles. By comparing the ZnO CTLs annealed in O_2_ or N_2_ with the as-deposited one, it is concluded that the oxygen vacancy (V_O_)-related defects dominate the bipolar programming characteristics of the TFT memory devices. For programming at positive gate voltage, electrons are injected from the IGZO channel into the ZnO layer and preferentially trapped at deep levels of singly ionized oxygen vacancy (V_O_^+^) and doubly ionized oxygen vacancy (V_O_^2+^). Regarding programming at negative gate voltage, electrons are de-trapped easily from neutral oxygen vacancies because of shallow donors and tunnel back to the channel. This thus leads to highly efficient erasing by the formation of additional ionized oxygen vacancies with positive charges.

## Background

A thin-film transistor (TFT) based on amorphous indium–gallium–zinc–oxide (a-IGZO) has been extensively studied for the application to flexible and transparent electronic systems [1–12]. This is attributed to some specific properties of a-IGZO films such as good uniformity, low processing temperature, visible light transparency, and high electron mobility [13]. Other than that, a-IGZO TFT nonvolatile memories have also been proposed, and its nonvolatile data storage capability expands the scope of the a-IGZO TFT device utilization. As a typical architecture of nonvolatile memory devices, a floating-gated a-IGZO TFT memory has been intensively investigated in recent years. Up to now, various materials have been explored as a floating gate (i.e., charge storage medium), such as dielectrics [14, 15], metal nanocrystals [16, 17], and semiconducting materials [18–21]. Since a-IGZO is a natural n-type semiconductor, and hole inversion is hardly realized in an a-IGZO TFT under a negative gate bias, therefore, the a-IGZO TFT memories usually have a poor erasing efficiency. In other words, most a-IGZO TFT memories cannot be electrically erased through hole injection from the channel [14–16]. Nevertheless, Zhang et al. [21] fabricated a TFT memory using a-IGZO as both the charge trapping layer (CTL) and the channel layer, which exhibited electrically programmable and erasable characteristics, as well as good data retention. Meanwhile, Yun et al. also investigated the characteristics of the a-IGZO TFT memories with different compositional IGZO CTL, revealing a decreasing memory window with increasing the O_2_ partial pressure (P_O2_) during sputtering deposition of the CTL [18]. In addition, Bak et al. reported the performance of the a-IGZO TFT memories with various conductivity ZnO CTLs and inferred that the optimized electronic nature of bandgap structure for the ZnO CTL could be one of the most important factors to realize highly functional oxide TFT memories [20]. Although the aforementioned oxide semiconductor CTL-based a-IGZO TFT memories exhibit superior electrical programming/erasing speeds, the bipolar programming characteristics of the abovementioned devices have not been reported, and the corresponding capture processes of different charges in the CTL of oxide semiconductor are not clear yet, especially for the trapping of positive charges.

In this work, a bipolar programmable a-IGZO TFT memory was fabricated by using an atomic-layer-deposited ZnO film as a CTL. By comparing the bipolar programming characteristics of the TFT memory devices with the as-deposited, O_2_- or N_2_-annealed ZnO CTLs, the capture processes of different charges in the ZnO layer were discussed. It is revealed that oxygen vacancy-related defects dominate the bipolar programming characteristics of the a-IGZO TFT memory devices.

## Methods

P-type Si (100) wafers with resistivity of 0.001–0.005 Ω cm were cleaned using the standard RCA cleaning process and used as the back gate of the device. Then, 35-nm Al_2_O_3_ and 20-nm ZnO films were deposited successively by atomic layer deposition (ALD) at 250 °C and 200 °C, which served as the blocking layer and CTL of the TFT memory, respectively. It is worth mentioning that the ZnO film has a root–mean–square (RMS) roughness of 0.553 nm. Subsequently, photolithography and wet etching were performed to define the CTL of ZnO. After that, an 8-nm Al_2_O_3_ tunneling layer was grown by ALD. The precursors of diethylzinc (DEZ)/H_2_O and TMA/H_2_O were used for the growth of ZnO and Al_2_O_3_ films, respectively. Thereafter, a 40-nm a-IGZO film was deposited by radio frequency magnetron sputtering as a channel layer at room temperature by using an InGaZnO_4_ target. The active channel with a width (W)/length (L) of 100/10 μm was then defined by photolithography and diluted HCl etching. Source and drain contacts of Ti/Au (30 nm/70 nm) were formed by e-beam evaporation followed by a lift-off process. Finally, all the fabricated devices were annealed at 250 °C in O_2_ for 5 min to improve its performance. The electrical characterizations were performed by using a semiconductor parameter analyzer (Agilent B1500A) at room temperature. The threshold voltage (V_th_) is defined as the gate voltage at which the drain current equals to W/L×10^−9^ A. The carrier concentration of ZnO films were extracted from Hall effect measurements (Ecopia HMS-3000) at room temperature.

## Results and Discussion

**Fig. 1:**
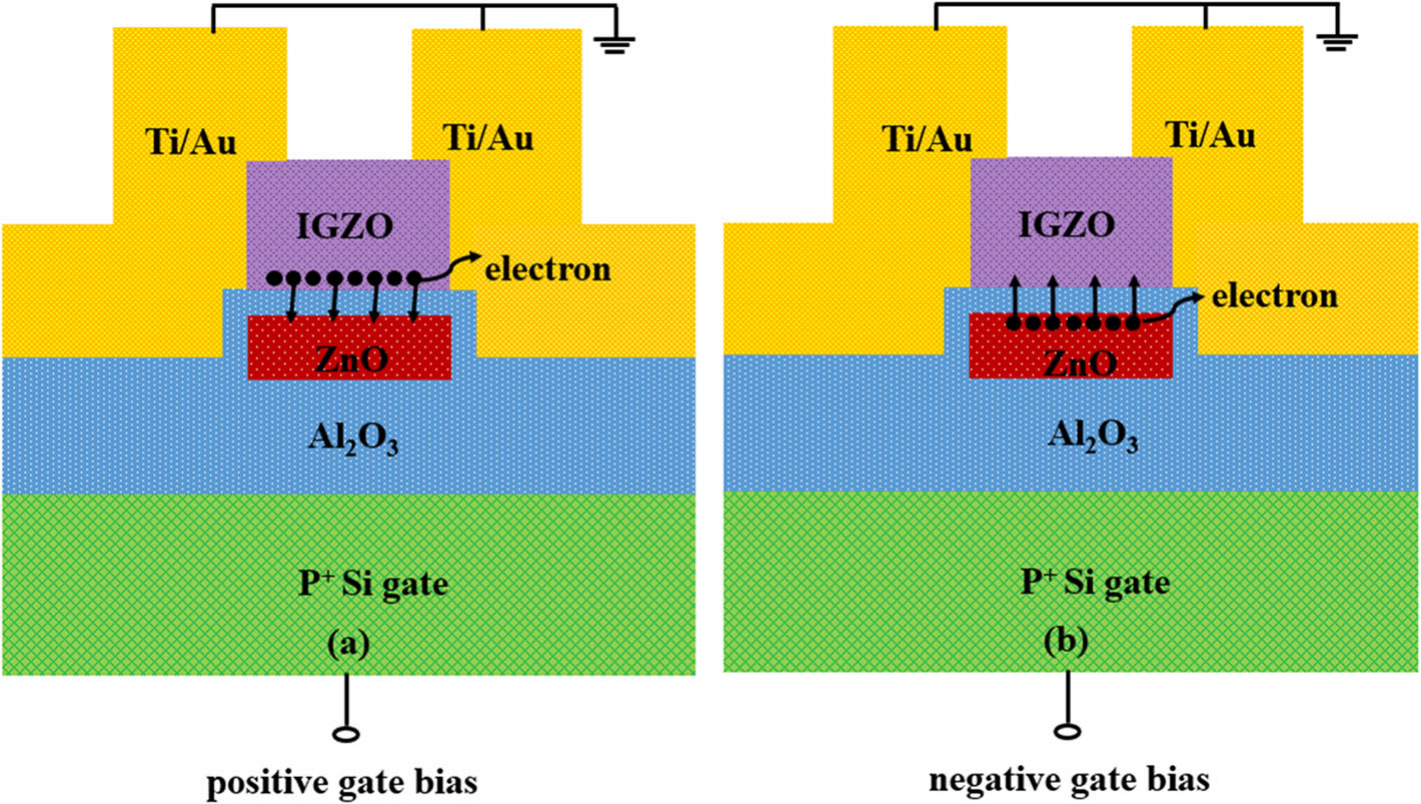
The cross section schematic diagrams of the a-IGZO TFT memory device programmed under a positive gate bias (**a**) and a negative gate bias (**b**), respectively.

**Fig. 2:**
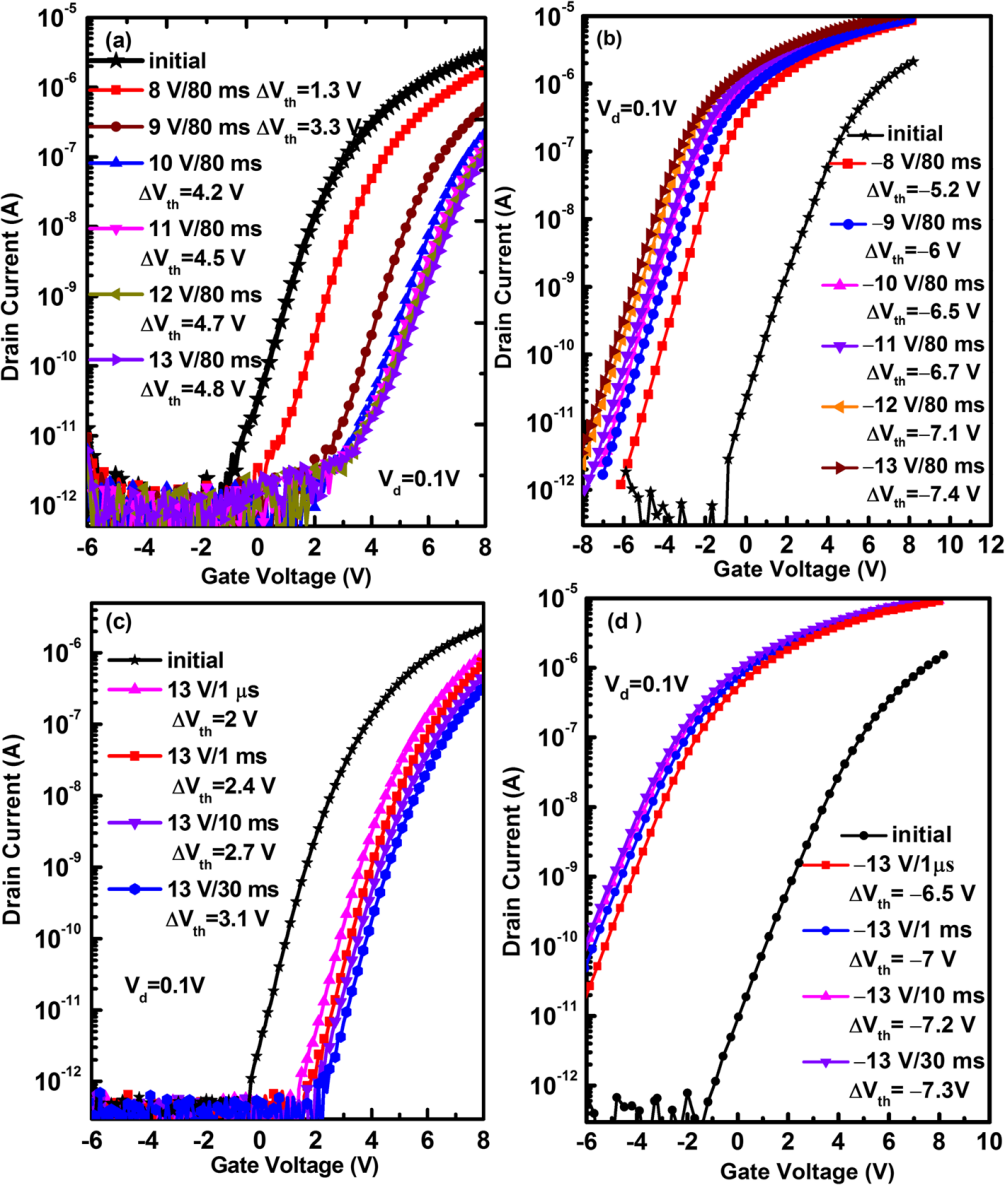
The transfer curves of the a-IGZO TFT memory device and those programmed **a** at various positive gate biases for a constant time of 80 ms, **b** at various negative gate biases for a constant time of 80 ms, **c** at 13 V for various programming time, and **d** at −13 V for various programming time. All the transfer curves for each figure were measured on the same device, and all programming operations were carried out in sequence.

Figure 1 shows the schematic diagrams of the fabricated a-IGZO TFT memory device under positive and negative bias programming, respectively. During electrical programming, an electrical pulse is applied on the back gate, and the source and drain electrodes are grounded. Figure 2 shows the programming characteristics of the pristine memory devices under different conditions. For the pristine memory device, it exhibits an on/off current ratio (*I*_on_/*I*_off_) of 1.5 × 10^7^, field-effect mobility of 7.1 cm^2^ V^−1^ s^−1^, and a subthreshold swing (SS) of 0.67 V/dec. In terms of 80 ms programming at different positive biases, the *I*_d_–*V*_g_ curve moves gradually in the direction of a positive bias as a function of programming voltage, e.g., the resulting V_th_ shift relative to the pristine device (ΔV_th_) increases from 1.3 to 4.8 V with increasing programming voltage from 8 to 13 V, exhibiting programming saturation at 12 V, as shown in Fig. 2a. Such a significant ΔV_th_ suggests that considerable electrons from the n-type a-IGZO channel are injected into the ZnO CTL. Moreover, when the programming voltage is fixed at 13 V, the ΔV_th_ increases slowly from 2 to 3.1 V with prolonging programming time from 1 μs to 30 ms, as shown in Fig. 2c. Interestingly, when the pristine memory device is programmed at a negative gate bias, the V_th_ exhibits a notable shift towards a negative bias, shown in Fig. 2b. For constant programming time of 80 ms, the ΔV_th_ enlarges from −5.2 to −7.4 V with increasing programming bias from −8 to −13 V. Even if the pristine memory device is programmed at -13 V for 1 μs, it can also demonstrates a ΔV_th_ as large as −6.5 V, shown in Fig. 2d. This means that a very large number of electrons are de-trapped from the CTL, hence resulting in remain of plenty of positive charges.

**Fig. 3:**
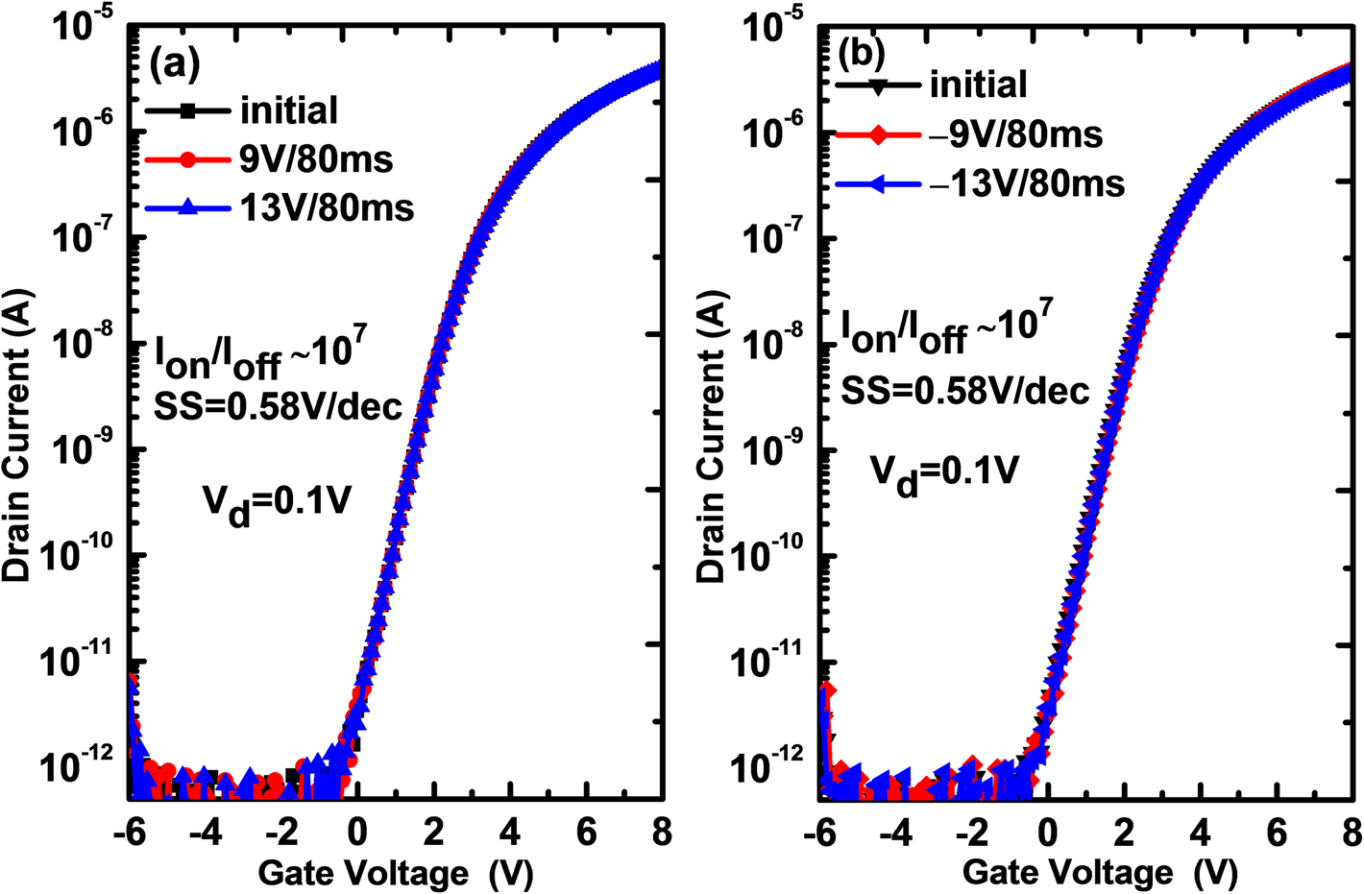
The transfer curves of the a-IGZO TFT device and those programmed **a** at different positive gate biases for a constant time of 80 ms and **b** at different negative gate biases for a constant time of 80 ms

**Fig. 4:**
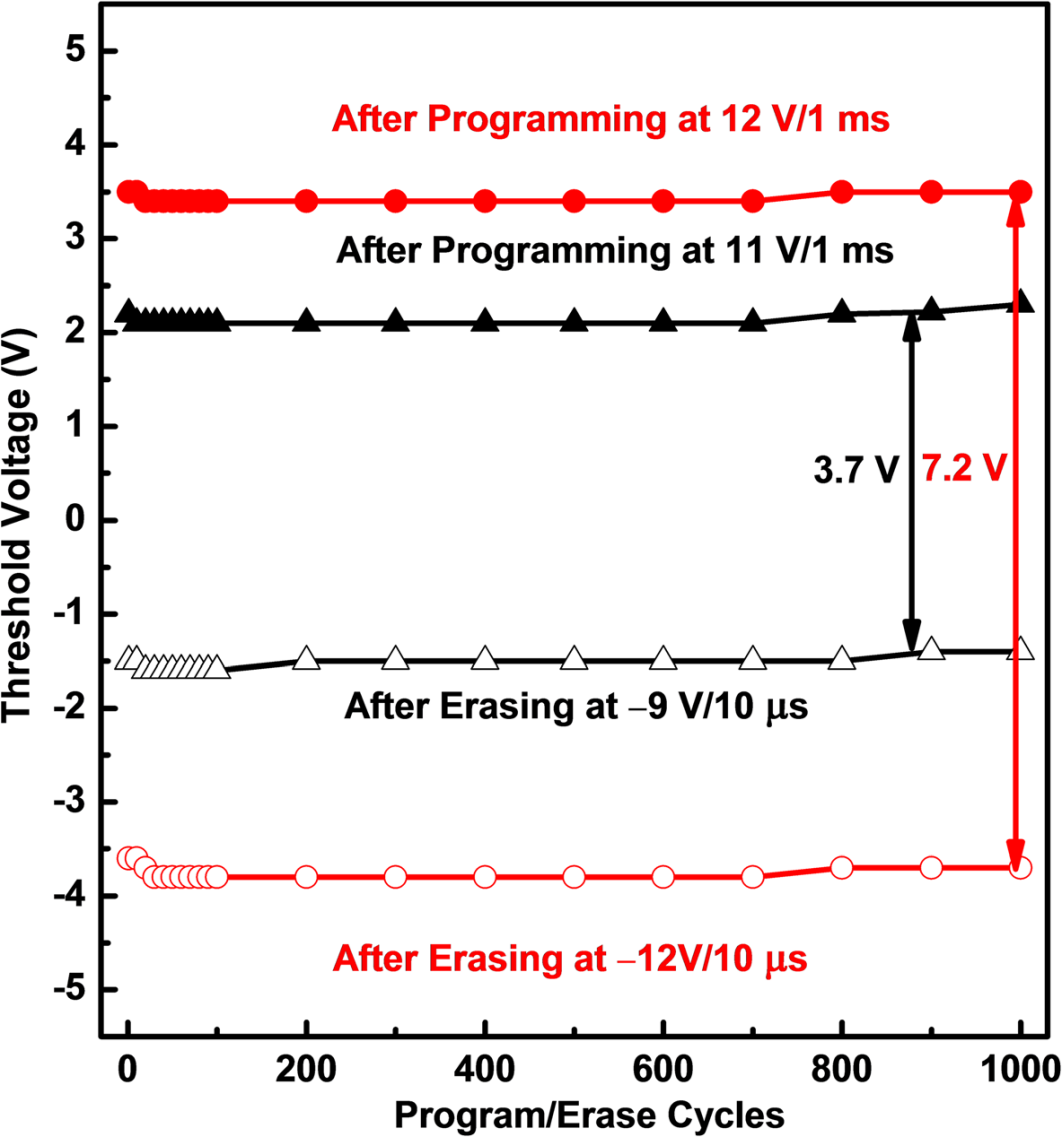
The endurance characteristics of the a-IGZO TFT memory device as a function of P/E cycles

To understand the charge trapping effect of the ZnO layer, a-IGZO TFTs without ZnO CTL are also fabricated as control devices for comparison. Figure 3 shows the transfer characteristics of the control devices when being programmed under different positive and negative biases, respectively. It is found that the device does not exhibit a discernible ΔV_th_ regardless of programming voltage polarity and amplitude. This indicates that the aforementioned distinct ΔV_th_ for the memory devices should be ascribed to the ZnO CTL. On the other hand, it is noted that IGZO is a natural n-type semiconductor, thus electrons in the IGZO channel can be easily injected into the ZnO CTL under a positive gate bias (e.g*.*, +9 V). However, when a negative programming bias is applied to the gate electrode of the device, the a-IGZO channel tends to be depleted, and the hole conduction is hardly achieved [15]. In this case, the device cannot be programmed via hole injection from the channel, and thus the unique possibility of electrical programming is to be realized by de-trapping of intrinsic electrons in the pristine ZnO CTL. In fact, our experimental results indicate that the device can be easily programmed under negative gate biases, see Fig. 2d. Figure 4 shows the endurance characteristics of the memory as a function of programming/erasing (P/E) cycles. The device exhibits a memory window of 3.7 V for 10^3^ of P/E cycles in the case of 11 V/1 ms programming and −9 V/10 μs erasing. Further, a memory window as large as 7.2 V can be achieved at 10^3^ of P/E cycles with respect to 12 V/1 ms programming and −12 V/10 μs erasing. Table 1 compares the programming and erasing characteristics of various a-IGZO TFT memories [14, 22, 23]. Compared to other devices, our device exhibits a much higher erasing efficiency even under a lower bias (−12 V) and much shorter time (10 μs) in spite of not notable superiority in programming efficiency.

**Table 1:** Comparisons of the programming and erasing characteristics of various a-IGZO TFT memories with different gate stacks

Gate stack	Programming conditions	ΔV_th-P_	Erasing conditions	ΔV_th-E_	Ref.
Al_2_O_3_/Al_2_O_3_/Al_2_O_3_	Vg = 10 V/10 ms	2 V	−10 V/100 s	−0.2 V	[14]
SiO_2_/SmTiO_3_/SiO_2_	Vg = 15 V/100ms	2.7 V	−15 V/100 ms	−2.2 V	[22]
SiO_2_/ErTi_x_O_y_/SiO_2_	Vg = 20V/100 ms	3.9 V	−20 V/100 ms	−3.9 V	[23]
Al_2_O_3_/ZnO/Al_2_O_3_	Vg = 13 V/1 μs	2 V	−12 V/10 μs	−7.2 V	This work

ΔV_th-P_ means the threshold voltage shift relative to the pristine device after programming and ΔV_th-E_ implies the threshold voltage shift relative to the programmed device after erasing

**Fig. 5:**
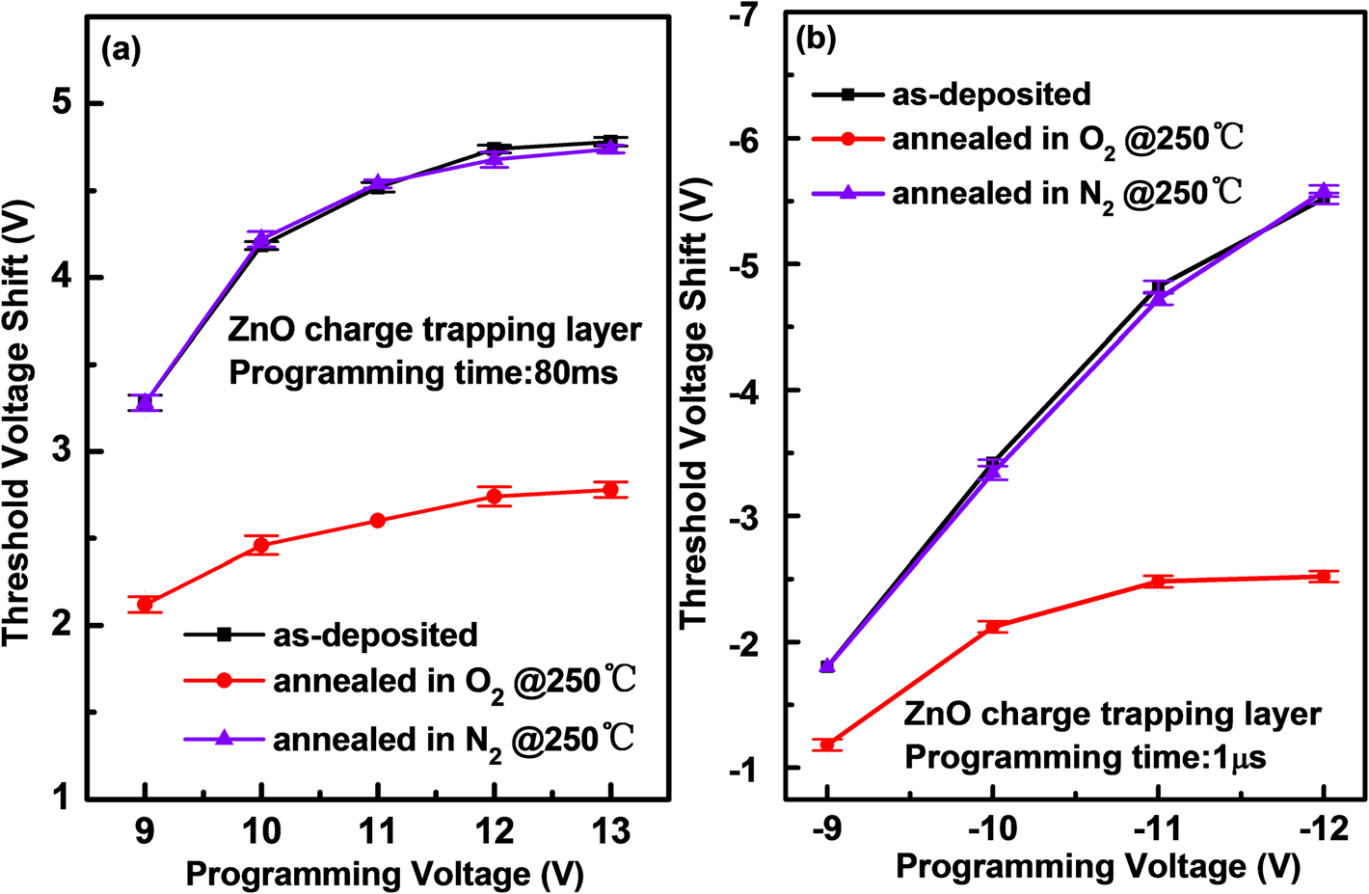
The threshold voltage shifts of the a-IGZO TFT memory devices with different processed ZnO charge trapping layers as a function of **a** positive programming voltage for constant programming time of 80 ms and **b** negative programming voltage for constant programming time of 1 μs. For each condition, five devices were measured.

To clarify the origin of electrons de-trapped from the pristine ZnO CTL, various processed ZnO CTLs are compared in the a-IGZO TFT memory devices. Figure 5 shows the programming voltage dependence of △V_th_ for the devices with different ZnO CTLs. It is observed that, for the memory devices with the as-deposited and N_2_-annealed ZnO CTLs, the resulting ΔV_th_ exhibits a similar increasing tendency with raising programming voltage despite of voltage polarities. However, for the memory device with the O_2_-annealed ZnO CTL, the absolute value of ΔV_th_ shows a significant decrease under the same programming condition, e.g., the absolute value of ΔV_th_ decreases by 2 and 3 V, respectively, in the case of 13 V/80 ms and −12 V/1 μs programming pulses. Furthermore, saturated programming behaviors are observed for the O_2_-annealed ZnO CTL in the case of positive and negative gate biases. This should be ascribed to limited traps in the CTL. In a word, the post-annealing in O_2_ at 250 °C reduces the number of trap centers in the ZnO film, hence leading to a decrease in charge trapping capacity.

**Fig. 6:**
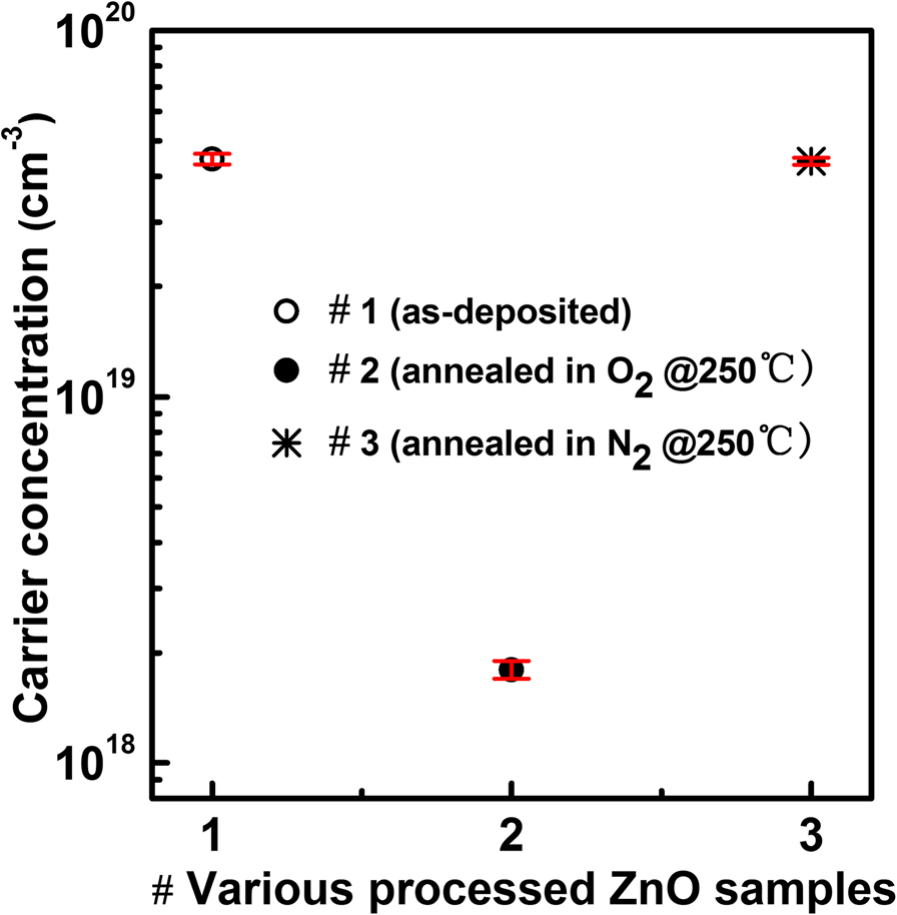
The carrier concentrations of the as-deposited ZnO film and those annealed under different conditions.

**Fig. 7:**
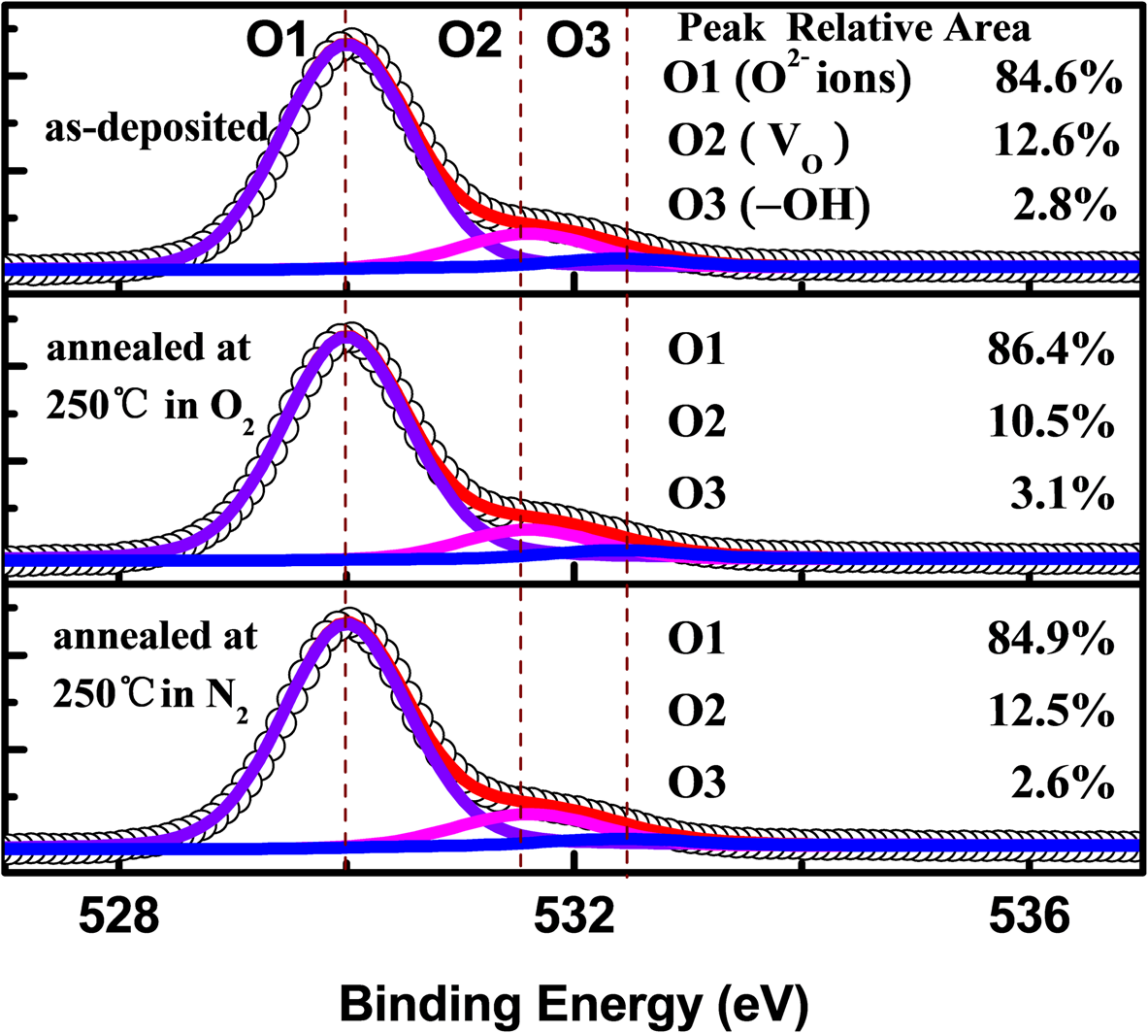
High-resolution O1s XPS spectra of the as-deposited ZnO film and those annealed at 250 °C in O_2_ and N_2_, respectively. O1 and O2 correspond to O^2−^ ions bound with Zn^2+^ and oxygen vacancies, respectively. O3 is attributed to chemisorbed oxygen element (–OH, etc). To remove unintentionally surface contaminants, all the samples were etched with in situ Ar ion bombardment

To investigate the influence of post-annealing on the properties of the ZnO film, the as-deposited and processed ZnO films are characterized by Hall effect measurements and XPS. As shown in Fig. 6, the ZnO film annealed in N_2_ at 250 °C shows a carrier concentration of 4.4×10^19^ cm^−3^, which is very close to that (4.5 × 10^19^ cm^−3^) of the as-deposited ZnO film; however, the ZnO film annealed in O_2_ at 250 °C exhibits a remarkable decrease in carrier concentration, which is equal to 1.8 × 10^18^ cm^−3^. It is reported that the intrinsic donors in n-type ZnO semiconductor films are oxygen vacancies [24]. Kwon et al. also reported that the O/Zn atomic ratio in the ALD ZnO film decreased gradually from 0.90 to 0.78 with increasing the deposition temperature from 70 to 130 °C [25]. This reveals the existence of oxygen vacancies in ALD ZnO films. Therefore, the O_2_-annealing-induced decrease in carrier (electron) concentration should be related to the reduction of oxygen vacancies in the ZnO film. Further, high-resolution O1s XPS spectra of the as-deposited ZnO film and those annealed in N_2_ or O_2_ are analyzed, as shown in Fig. 7. The deconvoluted three peaks are centered at 530.0, 531.6, and 532.4 eV, corresponding to O^2−^ ions bound with Zn^2+^ (O1), oxygen vacancies (O2), and chemisorbed oxygen element (–OH, etc.) (O3), respectively [26]. Compared with the as-deposited ZnO film, the post-annealing in O_2_ generates a decrease of 2.1% in the relative percentage of O2. Nevertheless, for the ZnO film annealed in N_2_, the relative percentage of O2 is almost unchanged. These results indicate that the O_2_ annealing can passivate oxygen vacancies in the ZnO film, but the N_2_ annealing cannot do. This further confirms the correlation between oxygen vacancies and carrier concentration.

**Fig. 8:**
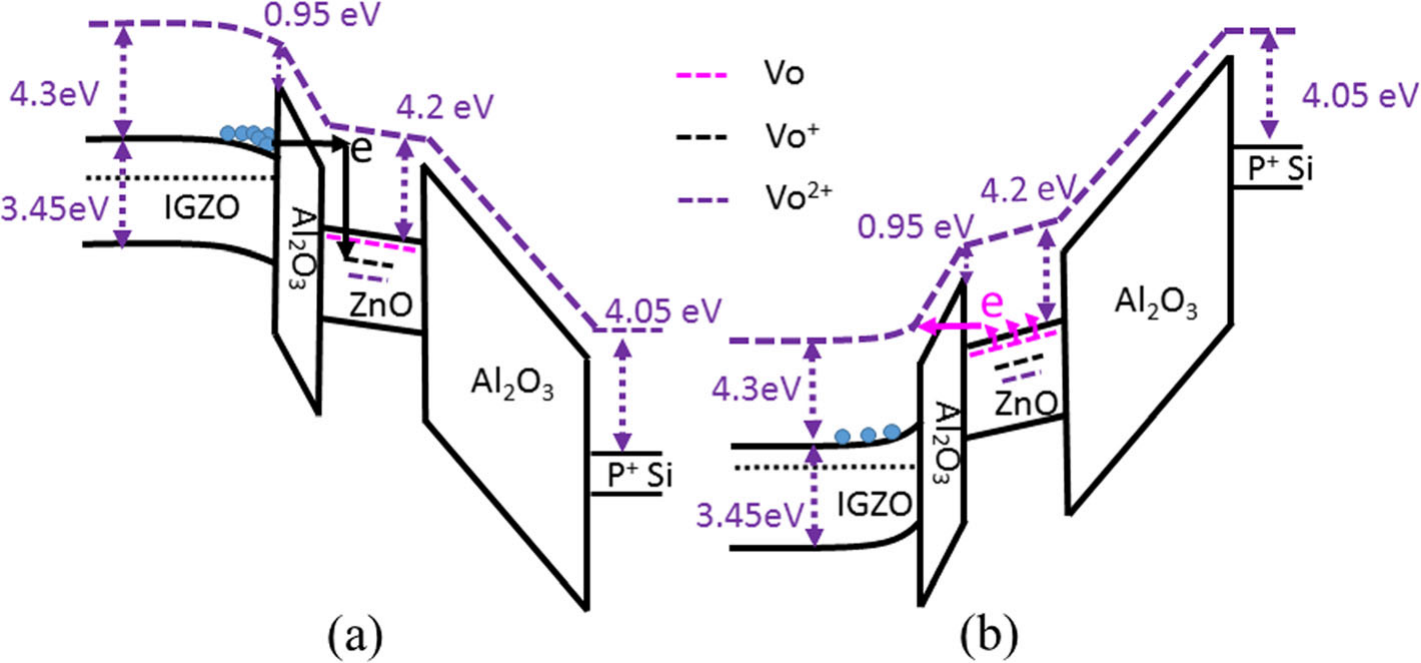
Energy band diagrams of the a-IGZO TFT memory devices programmed at **a** a positive gate bias and **b** a negative gate bias, respectively. V_o_, V_o_^+^, and V_o_^2+^ denote neutral oxygen vacancy, singly ionized oxygen vacancy, and doubly ionized oxygen vacancy, respectively

Based on the aforesaid experimental results, it can be concluded that the programming characteristics of the pristine memory devices are dominated by the concentration of oxygen vacancy-related defects in the ZnO CTL. In other words, oxygen vacancies in the ZnO film primarily serve as trap centers for trapping of positive and negative charges. It is reported that oxygen vacancy-related defects in ZnO include neutral oxygen vacancy (V_O_), singly ionized oxygen vacancy (V_O_^+^), and doubly ionized oxygen vacancy (V_O_^2+^), whose energy levels are located at 0.02–0.04, 0.3–0.45, and 0.61 eV, respectively, below the conduction band minimum of ZnO [27, 28]. Since the as-deposited ZnO film shows a high electron concentration in our case, the concentration of neutral oxygen vacancies serving as shallow donors should be much higher than that of ionized oxygen vacancies (V_O_^+^ and V_O_^2+^). In terms of programming at a positive gate bias, electrons in the accumulation layer of the a-IGZO channel are injected into the ZnO layer by the Fowler–Nordheim (F-N) tunneling mechanism, which is demonstrated by an incremental ΔV_th_ with enhancing programming voltage in Fig. 2a. Meanwhile, it is expected that these electrons are trapped preferentially at deep levels of V_O_^+^ and V_O_^2+^, as depicted in Fig. 8a. This causes a shift of V_th_ towards a positive bias. Of course, in addition to oxygen vacancies that trap electrons, other defects also could capture electrons. However, our experimental data indicate that oxygen vacancies play a crucial role in electron trapping as well as positive charge capture, as revealed in Fig. 5. Under negative programming voltage, the neutral oxygen vacancies in the pristine ZnO CTL dominantly donate electrons because of the shallowest energy level [27, 28], and the released electrons tunnel from the ZnO CTL into the channel, hence leading to the formation of positively charged oxygen vacancies (e.g., V_O_^+^), as shown in Fig. 8b. This causes a shift of V_th_ in the direction of negative bias, as indicated in Fig. 2b. Further, owing to a higher concentration of neutral oxygen vacancies (V_O_) in the as-deposited CTL of ZnO, the pristine memory device exhibits a much higher programming efficiency under the negative gate bias than under the positive gate bias. For example, the absolute value of ΔV_th_ is as large as 6.5 V after programming at −13 V for 1 μs (see Fig. 2d); however, the ΔV_th_ is equal to 2 V after programming at 13 V for 1 μs (Fig. 2c). This is because the former is determined mainly by the concentration of V_O_, and the latter is dominated by the concentrations of V_O_^+^ and V_O_^2+^.

## Conclusions

In summary, we fabricated a bipolar programmable a-IGZO TFT memory with an atomic-layer-deposited ZnO CTL. Compared with the programming under a positive gate bias, the programming under a negative gate bias can generate a much higher efficiency. This is because different oxygen vacancy defects take effect during voltage-polarity-dependent programming. That is, deep defects of V_O_^+^ and V_O_^2+^ play a key role for electrons trapping during positive bias programming, and shallow defects of V_O_ mainly donate electrons during negative bias programming, resulting in the generation of positively charged oxygen vacancies.

## Data Availability

The datasets supporting the conclusions of this manuscript are included within the manuscript.

## Abbreviations

a-IGZO: Amorphous indium-gallium-zinc-oxide
ALD: Atomic layer deposition
CTL: Charge trapping layer
TFT: Thin-film transistor
XPS: X-ray photoelectron spectroscopy
